# Ion‐Gated Nanoconfinement in Bimetallic Nanorattles Unlocks Enhanced Plasmonic Nitrate Reduction Electrocatalysis

**DOI:** 10.1002/smll.202507525

**Published:** 2025-09-12

**Authors:** Flavia G. da Silva, Kaline N. da Silva, Shiqi Wang, Hugo L. S. Santos, Eric V. Formo, Pedro H. C. Camargo

**Affiliations:** ^1^ Department of Chemistry University of Helsinki A.I. Virtasen aukio 1 Helsinki 00560 Finland; ^2^ Georgia Electron Microscopy University of Georgia Athens GA 30602 USA

**Keywords:** bimetallic nanostructures, electrocatalysis, nanoconfinement, nitrate reduction reaction (NO_3_RR), plasmonic enhancement

## Abstract

Nanoconfinement is a powerful strategy to modulate local reaction environments in electrocatalysis, yet direct experimental validation of its impact on multi‐electron transformations remains limited. Here, it is reported that hollow bimetallic Au@AgAu nanorattles serve as proof‐of‐concept tunable electrocatalysts for nitrate reduction (NO_3_RR). By varying the porosity of Ag‐based shells via controlled galvanic replacement, architectures are constructed in which electric double layer thickness, and thus pore accessibility, can be dynamically modulated and quantified by electrolyte concentration. With increasing ionic strength, a marked enhancement in NO_3_RR activity is observed, with nanorattles delivering three to fourfold higher mass‐specific currents than solid Au@Ag counterparts, attributable to expanded access to confined interior sites. Plasmonic excitation under visible light further amplifies performance under acidic conditions, highlighting a synergistic interplay between localized surface plasmon resonances and geometric confinement. Density functional theory calculations reveal that alloying and hollowing induce electronic delocalization, favorable charge redistribution, and optimized d‐band energetics. While activity and plasmonic enhancement are attenuated under alkaline conditions, the nanorattles still outperform solid analogues, demonstrating the broad utility of this design. Together, the findings establish a dynamic framework in which engineered porosity, bimetallic composition, and electrolyte‐driven surface accessibility converge to harness nanoconfinement for enhanced electrocatalysis.

## Graphical abstract

Hollow bimetallic Au@AgAu nanorattles with tunable porosity enable dynamic control of nanoconfinement via electrolyte concentration, boosting nitrate reduction activity up to fourfold over solid counterparts. Enhanced access to interior sites and plasmonic excitation under visible light synergistically improve performance, offering a versatile strategy to couple pore accessibility, composition, and light–matter interactions for advanced electrocatalysis.

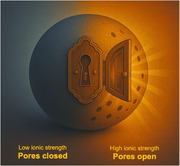

## 1 Introduction

Electrocatalysis is crucial for transforming renewable electricity into value‐added chemical products and mitigating environmental pollutants.^[^ ^1^ ^]^ In this context, the electrochemical reduction of nitrate (NO_3_RR) to ammonia, for example, has garnered considerable attention.^[^ ^2^, ^3^ ^]^ On the one hand, ammonia serves as a pivotal industrial feedstock and a promising energy carrier; on the other, reducing nitrate (a widespread water contaminant) can aid in environmental remediation.^[^ ^4^ ^]^ Among the various approaches to enhance NO_3_RR, nanoparticles with controlled morphologies, such as hollow interiors or porous shells, have shown promise in promoting multi‐electron pathways and boosting conversion rates.^[^ ^5^ ^]^ Nanoconfinement, where catalysis is restricted to an enclosed or partially occluded reactive space, is increasingly recognized as an effective route toward optimizing activity, selectivity, and stability.^[^ ^6^, ^7^ ^]^ Yet, despite the promise of bimetallic compositions and even plasmonic catalysis, establishing explicit correlations between confined geometric environments, local reaction conditions, and catalytic outcomes remains challenging.

While many reports have probed how particle size, crystal facets, and surface composition impact electrochemical reactions, leveraging nanoscale voids and open shells imposes multiple challenges.^[^ ^8^, ^9^ ^]^ First, it can be difficult to disentangle the benefits of a confined environment from simpler effects such as increased surface area or altered electronic structure. Second, the electric double layer (EDL) formed within pores or hollow interiors significantly influences the local potential distribution, but controlling or monitoring the EDL remains nontrivial, particularly under practical operating conditions.^[^ ^10^, ^11^, ^12^ ^]^ Third, even for catalysts designed to exploit LSPR (localized surface plasmon resonance) or synergistic bimetallic interactions, deconvoluting the role of pore accessibility from other structural variables (e.g., relative metal loadings, morphological stability under reaction conditions) poses serious experimental complexities. Therefore, establishing a robust experimental framework that specifically quantifies nanoconfinement effects while preserving high catalytic performance is key to unlocking next‐generation electrocatalysts for NO_3_RR and beyond.

In this work, we address these challenges by developing a family of Au@AgAu nanorattles featuring a hollow interior and porous Ag‐based shell, whose structural characteristics are finely tuned via partial galvanic replacement. This architecture is employed as a model system as it enables precise modulation of both shell porosity and internal void volume, key parameters for investigating nanoconfinement effects. By systematically varying the electrolyte concentration, we dynamically adjust the thickness of the EDL, thereby controlling the accessibility of interior catalytic surfaces. Through a combination of high‐resolution electron microscopy and UV–vis spectroscopy, we correlate morphological and compositional features with LSPR behavior and show that plasmonic excitation further enhances activity in acidic media. Electrochemical analyses reveal that more porous nanorattles deliver superior mass activity in NO_3_RR, with performance gains scaling with pore accessibility under high ionic strength conditions. Crucially, this electrolyte‐driven control enables us to isolate nanoconfinement effects from differences in morphology or Ag content. Complementary density functional theory calculations provide mechanistic insight, revealing that alloying and hollowing modulate electronic structure, enhance charge delocalization, and lower energetic barriers for key NO_3_RR steps. Together, our findings establish a framework in which geometric confinement, electronic tuning, and ion screening are integrated to unlock efficient and selective multi‐electron electrocatalysis.

## 2 Results and Discussion

**Figure 1:**
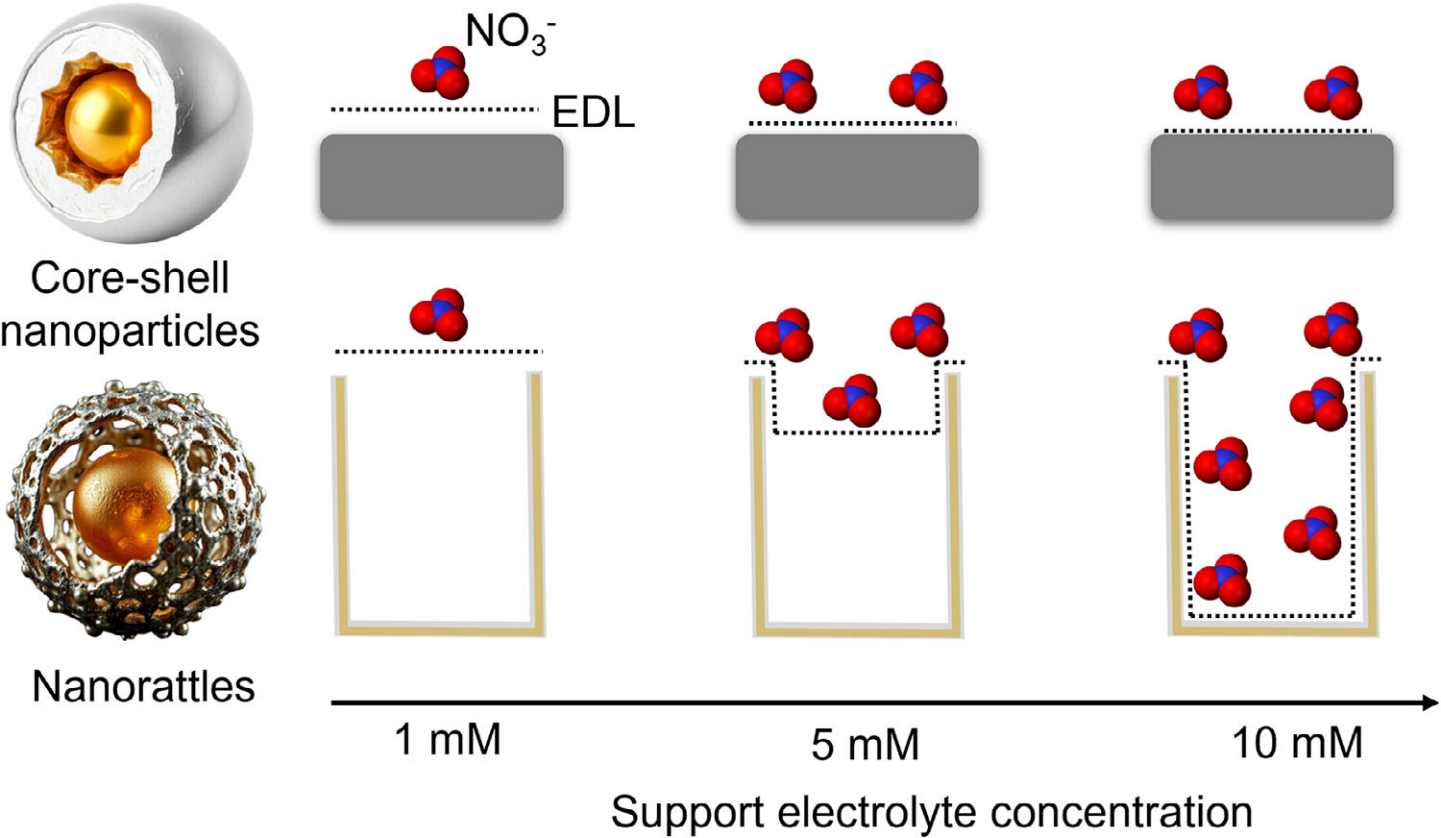
Schematic illustration of nanorattle‐based nanoconfinement in NO_3_RR. A solid Au@Ag core–shell nanoparticle (top left) is contrasted with a hollow Au@AgAu nanorattle (bottom left), where a porous AgAu shell surrounds an Au core, creating an internal void. Under lower electrolyte concentrations (left and middle columns), the EDL (dashed line) partially obstructs diffusion of nitrate molecules (red–blue spheres) into the interior of the rattles. In contrast, at higher ionic strengths (right column), the EDL shrinks, allowing enhanced access of nitrate to the catalytically active inner surfaces. This dynamic, ion‐driven modulation of pore accessibility underpins the role of nanoconfinement in boosting NO_3_RR electrocatalysis.

Building on our goal to investigate how engineered nanoconfinement influences the electrocatalytic NO_3_RR, we hypothesized that the hollow core and porous Ag‐based shell in Au@AgAu nanorattles would provide a confined environment that enhances catalytic activity, particularly when the accessibility of the inner surfaces is modulated by electrolyte concentration, as shown in **Figure** **1**. To probe this effect, we synthesized nanorattles with two distinct morphologies and porosities via a galvanic replacement reaction between AuCl_4_^−^ and Ag at the surface of Au@Ag core–shell nanoparticles, which served as templates. This method is advantageous as it allows precise control over the porous architecture by varying the AuCl_4_^−^ to Ag molar ratio during the replacement process. A higher AuCl_4_^−^ concentration promotes increased Ag dissolution, leading to a more pronounced hollowing effect and the formation of highly porous shells.^[^ ^13^, ^14^ ^]^ Following this approach, we synthesized two nanorattle compositions with differing extents of galvanic replacement, designated as Ag_61_Au_39_ and Ag_47_Au_53_, as determined by inductively coupled plasma atomic emission spectroscopy (MP‐AES, Table S1, Supporting Information).

**Figure 2:**
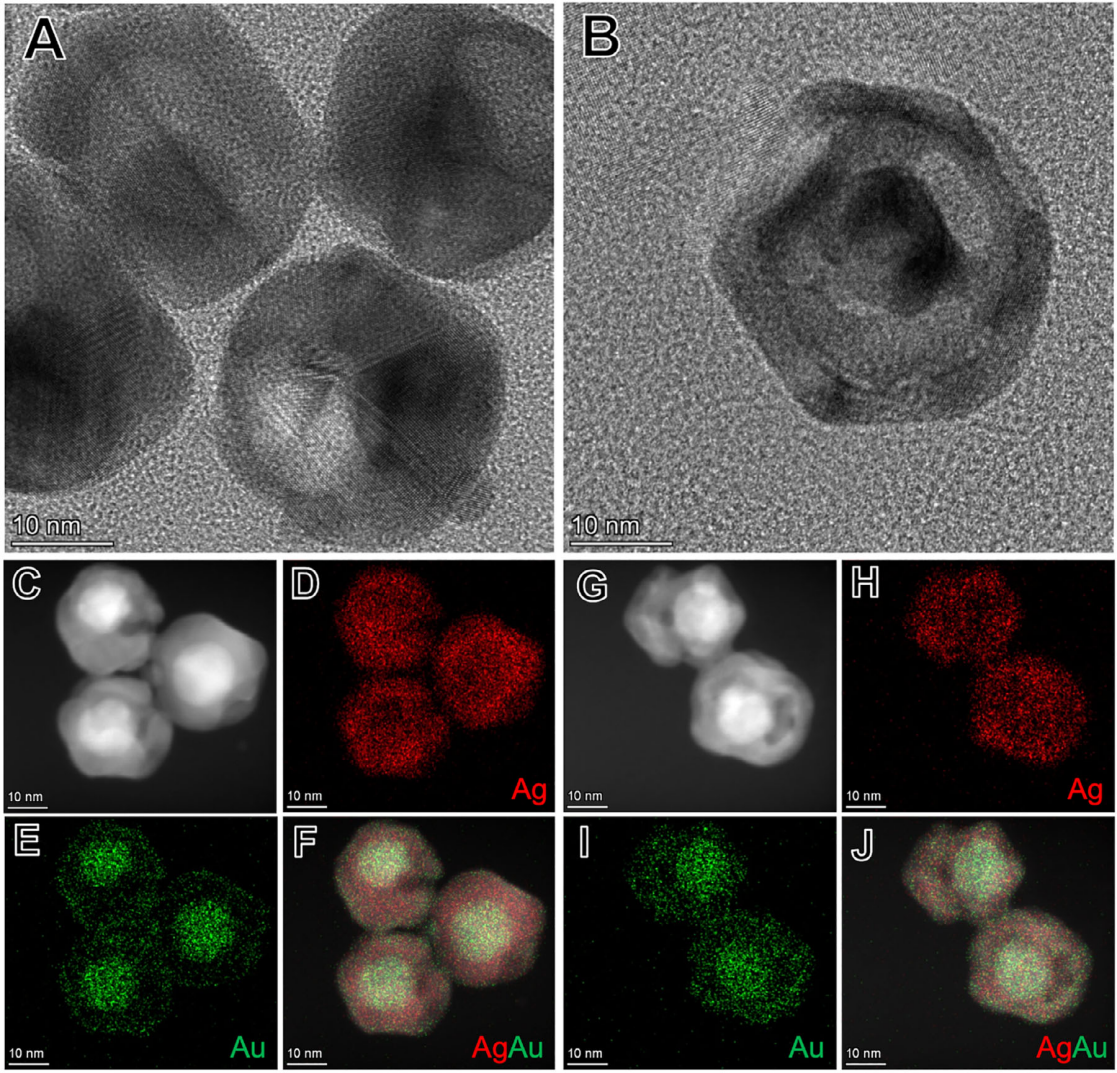
Structural and elemental characterization of bimetallic nanorattles. HRTEM images highlight the hollow nanorattle architecture of Ag_61_Au_39_ (A) and Ag_47_Au_53_ (B). The corresponding STEM–HAADF images (C,G) and STEM–EDS elemental maps for Ag (D,H), Au (E,I), and the Ag–Au overlay (F,J) confirm the pronounced porosity and bimetallic distribution. The Ag_61_Au_39_ sample (C–F) displays moderately porous walls with partial Ag dissolution, whereas the Ag_47_Au_53_ nanorattles (G–J) exhibit more extensive hollowing and higher Au content, consistent with greater galvanic replacement.

Figure S1 (Supporting Information) provides scanning electron microscopy (SEM) images for the Au@Ag core–shell nanoparticles employed as templates, along with SEM images of both nanorattle compositions, and corresponding size‐distribution histograms, whereas Figure S2 (Supporting Information) presents additional TEM images of the Au@Ag core–shell nanoparticles prior to galvanic replacement. All the particles presented quasi‐spherical shapes, and the average outer diameters corresponded to 27, 28, and 32 nm for Au@Ag core–shell nanoparticles, Ag_61_Au_39_, and Ag_47_Au_53_ nanorattles, respectively. High‐resolution transmission electron microscopy (HRTEM), high‐angle annular dark field (HAADF) scanning transmission electron microscope (STEM), and STEM energy‐dispersive X‐ray spectroscopy (STEM−EDS) elemental mapping of the Ag_61_Au_39_ and Ag_47_Au_53_ nanorattles are shown in **Figure** **2**. The HRTEM images (Figure 2A,B) confirm the successful fabrication of hollow nanorattle structures for both compositions, revealing a distinct core‐shell morphology with a visible interior void and a porous outer shell. For Ag_61_Au_39_ (Figure 2A), one observes a moderate level of Ag dissolution consistent with partial galvanic replacement, resulting in a shell with relatively few pores (around 1 pore per particle) surrounding a loosely defined void. By contrast, the Ag_47_Au_53_ nanorattles (Figure 2B) display a more pronounced porous shell structure with several pores and more extensive hollowing, consistent with the higher AuCl_4_^−^ concentration used during the synthesis. This greater porosity is expected to promote additional nanoconfinement effects, beneficial for electrocatalytic activity.^[^ ^15^ ^]^

STEM‐HAADF images and STEM‐EDS maps of Ag_61_Au_39_ (Figure 2C–F) and Ag_47_Au_53_ nanorattles (Figure 2G–J) further support these observations. The HAADF images (Figure 2C,G) highlight the core–shell contrast and thickness variations between the two samples: Ag_61_Au_39_ (Figure 2C) exhibits fewer dark regions in the shell (i.e., lower‐density), whereas Ag_47_Au_53_ (Figure 2G) shows a higher number of such voids, consistent with increased porosity. Both samples present a brighter core due to the presence of the Au. The corresponding STEM‐EDS maps (Figure 2D–F for Ag_61_Au_39_ and **H–J** for Ag_47_Au_53_) confirm that Ag remains heavily concentrated in the shell, while Au is enriched in the interior and, to a lesser extent, at the shell surface. Notably, the Ag_47_Au_53_ nanorattles show stronger Au signals, reflecting more extensive galvanic replacement, whereas Ag_61_Au_39_ retains a higher Ag content. The overlay maps (Figure 2F,J) further reveal a spatial gradient from an Au‐rich core to the Ag‐rich inner shells, and outer layer containing Au and Ag in Ag_61_Au_39_ (Figure 2F), versus a more uniformly mixed outer shell in Ag_47_Au_53_. Figure S3 (Supporting Information) provides additional HRTEM and STEM–HAADF images for both nanorattle compositions. Altogether, these data demonstrate that varying the ratio of galvanic replacement successfully modulates both porosity and elemental gradients, laying the foundation for understanding how such structural distinctions can translate into enhanced NO_3_RR.

Following our structural and compositional analyses, we turned to UV–vis absorption spectroscopy to explore how the plasmonic properties of these bimetallic nanostructures might be influenced by their composition and hollow architecture. As shown in Figure S4 (Supporting Information), the maximum of the extinction band was centered at 520 nm, 405 nm, 440 nm, and 512 nm for Au, Au@Ag, Ag_61_Au_39_, and Ag_47_Au_53_, respectively. The LSPR band at 520 nm is characteristic of the dipolar mode in Au NPs 15 nm in diameter.^[^ ^16^ ^]^ Upon Ag deposition at the surface to generate the Au@Ag core–shell NPs, the main LSPR band shifts to shorter wavelengths and becomes slightly broader, reflecting both the altered dielectric environment around the Au core and the emergence of Ag‐specific plasmonic contributions.^[^ ^17^ ^]^ In contrast, the nanorattles (Ag_61_Au_39_ and Ag_47_Au_53_) exhibit absorptions that span a wider spectral range, suggesting more complex plasmonic coupling within their hollow interiors and between the two metallic components. In particular, the higher‐Au‐content nanorattles show a discernible red shift in their dominant absorption, consistent with the formation of bimetallic compositions at the shell due to Au deposition, hollowing, and stronger coupling between shell and core components (plasmonic coupling).^[^ ^14^, ^18^ ^]^ These UV–vis results, together with the electron microscopy data, underscore how composition and morphology tune the plasmonic behavior, setting the stage for subsequent investigations into plasmon‐enhanced electrocatalytic performance.

Following the comprehensive structural and optical characterization of the Au–Ag nanorattles, which confirmed their distinct morphologies, porosities, and plasmonic properties, we next evaluated their catalytic performance in the NO_3_RR. We aimed to elucidate how the unique confinement within these hollow bimetallic architectures influences activity, selectivity, and stability under both acidic and alkaline conditions.

**Figure 3:**
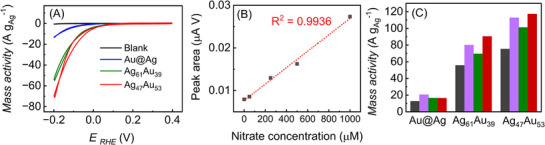
NO_3_RR under acidic conditions and plasmonic excitation. A) Cyclic voltammograms (CVs) measured in 0.1 m HClO_4_ at 25 °C and 0.01 V s^−1^ for the blank electrolyte (black line) and 10 mM NaNO_3_ on Au@Ag (blue), Ag_61_Au_39_ (green), and Ag_47_Au_53_ (red) in dark conditions, highlighting the enhanced activity of the nanorattles. B) Linear correlation between nitrate concentration and reduction peak area for Ag_47_Au_53_, demonstrating the catalyst's sensitivity toward nitrate. C) Chronoamperometric current densities recorded at −0.2 V under dark (black) and visible‐light irradiation (427 nm, purple; 525 nm, green; 640 nm, red), underscoring the plasmon‐driven boost in NO_3_RR.

**Figure** **3**A presents cyclic voltammograms (CVs) normalized by the mass of Ag recorded in acidic medium (0.1 mol L^−1^ HClO_4_) in the presence of nitrate for the Ag_61_Au_39_, and Ag_47_Au_53_ nanorattles (green and red traces, respectively). The data for Au@Ag nanoparticles (blue trace) as a control sample and for the pure electrolyte without nitrate (blank, black trace) are also included. Under these conditions, Ag corrosion seems negligible (Figure S5, Supporting Information). The onset potential for the NO_3_RR is approximately 0.1 V versus reversible hydrogen electrode (RHE) for all catalysts, regardless of structural differences. It is important to note that Ag is the primary electroactive species in this system, as Au shows negligible activity for NO_3_RR or NO_2_RR under our employed conditions (as described later in Figure S7, Supporting Information). All current densities are normalized to the Ag loading in the electrode to facilitate comparisons.^[^ ^19^, ^20^ ^]^ The CVs normalized by geometric area are shown in Figure S5 (Supporting Information).

Despite exhibiting the same onset potential, the voltammetric analysis revealed an exponential increase in current density up to −0.2 V versus RHE. To avoid interference from the hydrogen evolution reaction (HER), more negative potentials were excluded. Notably, the nanorattle catalysts (Ag_61_Au_39_ and Ag_47_Au_53_) displayed significantly enhanced NO_3_RR activity compared with the core–shell Au@Ag nanoparticles, achieving current densities of 13.4 A g^−1^ (Au@Ag NPs), 53.5 A g^−1^ (Ag_61_Au_39_), and 69.3 A g^−1^ (Ag_47_Au_53_). This corresponds to a three‐ to fourfold improvement for the nanorattles over the core–shell catalyst, reflecting how the hollow interior and highly porous Ag–Au shell effectively increases the accessible surface area for nitrate adsorption and subsequent reactions.

To further support the enhanced catalytic performance of the nanorattle structures, we measured the double‐layer capacitance (C_dl_) as a proxy for electrochemical surface area (ECSA) of each catalyst using cyclic voltammetry in the non‐faradaic region at various scan rates (Figure S6, Supporting Information). The C_dl_ values, normalized by Ag mass, followed the order Ag_61_Au_39_ (4.3 mF mg^−1^) > Ag_47_Au_53_ (3.7 mF mg^−1^) ≫ Au@Ag (1.6 mF mg^−1^). These results confirm that the hollow and porous morphology of the nanorattles exposes significantly larger electroactive areas than the solid core–shell counterpart. This increase in ECSA directly contributes to the several‐fold increase in current densities shown in Figure 3A, demonstrating that the superior activity arises not only from composition but also from the structural advantages of the nanorattle design.

To clarify the roles of Au and Ag in NO_3_RR activity, we performed control experiments with pure Au and pure Ag nanoparticles under identical conditions (Figure S7, Supporting Information). Au nanoparticles exhibited negligible activity, whereas Ag nanoparticles showed measurable but substantially lower activity than both the Au@Ag core–shell and the nanorattle catalysts. These results confirm that, although Ag provides the primary active sites, the incorporation of Au is essential for achieving superior performance.

Figure 3B shows the linear dependence of the reverse‐scan reduction peak area on [NO_3_^−^] after incremental nitrate additions (Figure S8, Supporting Information) for the Ag_47_Au_53_ nanorattles. A strong linear correlation (R^2^  =  0.9936) indicates that these catalysts offer a highly sensitive response, highlighting their potential for quantitative NO_3_^−^ detection in acidic media. In the studied NO_3_^−^ concentration range (up to 1 mm; Figure 3B; Figure S8, Supporting Information), the reduction peak area increased linearly with concentration, with no indication of saturation under our experimental conditions. This linearity suggests that the catalytic sites within the nanorattles remain accessible and active across this range, likely due to dynamic pore accessibility modulated by electrolyte concentration. In addition to increased activity, chronoamperometry over 250 min revealed a 7% activity loss for Ag_47_Au_53_ nanorattles compared with a 34% decline observed for the core–shell structure (Figure S9, Supporting Information), underscoring the superior stability imparted by the nanorattle architecture in acidic media.

We next investigated the NO_3_RR under visible‐light irradiation to harness plasmonic effects from both Au and Ag. Figure 3C highlights the mass activity at −0.2 V versus RHE under illumination at 427 nm, 525 nm, and 640 nm. In all cases, the electrocatalytic activity increased markedly compared with dark conditions, consistent with plasmonic excitation in Au and Ag that generates hot carriers and localized heating to enhance reaction rates.^[^ ^14^ ^]^ For Au@Ag nanoparticles, the mass activity rose from 12.6 A g^−1^ (dark) to 20.5, 16.6, and 16.4 A g^−1^ at 427, 525, and 640 nm, corresponding to improvements of 62%, 31%, and 31%, respectively. Even more pronounced enhancements were observed with Ag_61_Au_39_ (from 55.7 A g^−1^ in the dark to 80, 70, and 90 A g^−1^, respectively) and Ag_47_Au_53_ (75.3 A g^−1^ in the dark, rising to 112.8, 101.2, and 117.3 A g^−1^ under illumination). These results indicate maximum enhancements of up to 61.6% (Ag_61_Au_39_) and 56.4% (Ag_47_Au_53_) upon light irradiation. Current densities normalized by geometric areas are presented in Figure S10 (Supporting Information).

Reaction kinetics investigations (Figure S11, Supporting Information) indicated a near‐unity reaction order with respect to nitrate concentration, underscoring its pivotal role in determining the overall rate. Because nitrite (NO_2_^−^) is widely recognized as the primary intermediate formed after the rate‐determining step in NO_3_RR, we substituted nitrate with nitrite in acidic solution (Figure S12, Supporting Information) to further probe the mechanistic pathways.^[^ ^21^ ^]^ Unlike nitrate reduction reaction, which begins near 0.1 V, linear voltammograms recorded from 0.4 to −0.2 V versus RHE showed reduction processes across the whole potential range. Under 640 nm illumination, the nitrite reduction current increased significantly, by 85%, 140%, and 126% for Au@Ag core–shell nanoparticles, Ag_61_Au_39_, and Ag_47_Au_53_, respectively, highlighting the strong influence of LSPR on NO_2_^−^ conversion. Further NO‐stripping analyses (Figure S13, Supporting Information) confirm that these currents cannot be attributed to NO reduction reaction, which initiates only below −0.1 V and likely proceeds via (NO)_2_ dimerization to form N_2_O.^[^ ^22^ ^]^ Although restricting potentials to −0.2 V may not fully remove all adsorbed NO, photoexcitation nonetheless boosts the accessible current, emphasizing the synergy between potential control and plasmonic activation.

**Figure 4:**
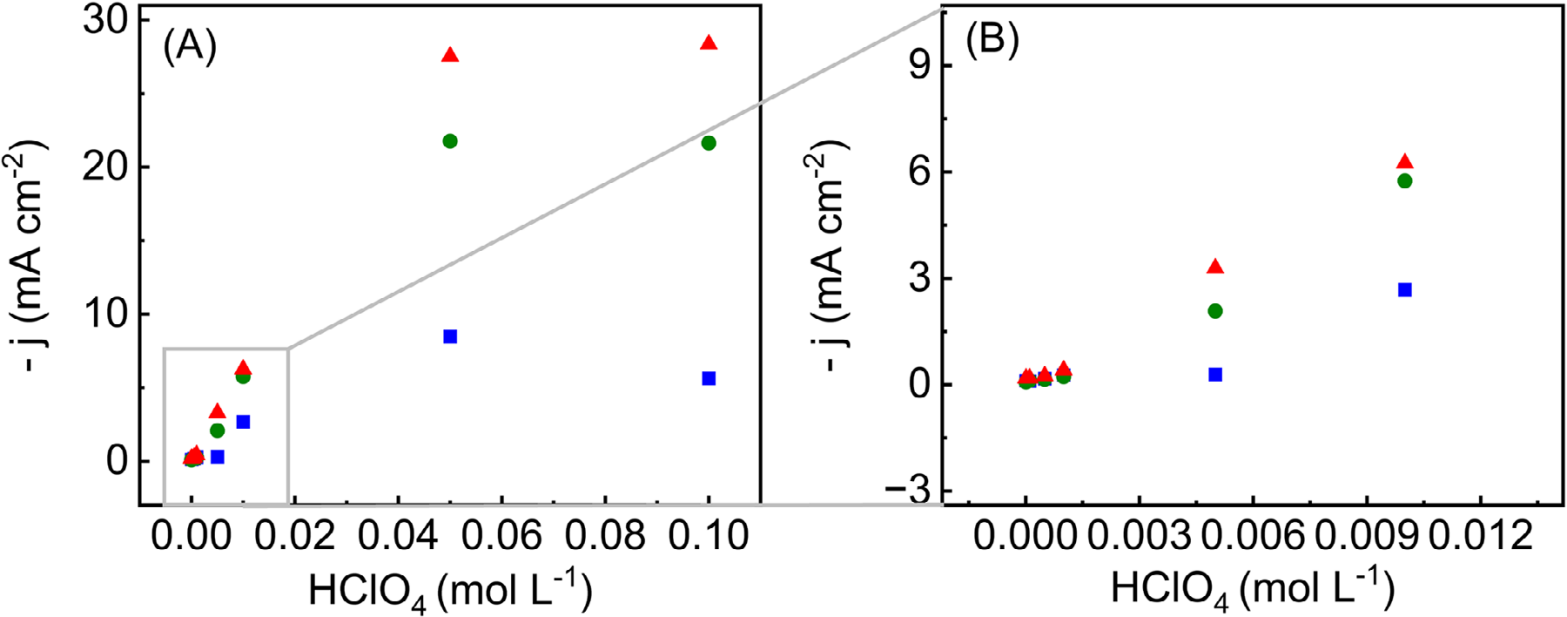
Electrolyte‐controlled nanoconfinement effects in NO_3_RR. A) Chronoamperometric currents at −0.2 V versus RHE are plotted against perchloric acid concentration for Au@Ag (blue squares), Ag_61_Au_39_ (green circles), and Ag_47_Au_53_ (red triangles) at 25 °C, illustrating a pronounced increase in NO_3_RR activity as ionic strength rises. B) Magnified view of the region in A (boxed area), highlighting early‐stage differences at lower acid concentrations. The strong response of the more porous nanorattles (green and red) underscores the critical role of nanoconfinement and EDL thinning in enhancing nitrate reduction, whereas the solid core–shell catalyst (blue) shows comparatively modest gains.

Building on the plasmonic and morphological insights discussed above, we next investigated how electrolyte concentration affects the accessibility of the nanorattles’ porous interiors, thus testing our central hypothesis regarding nanoconfinement control. **Figure** **4** shows the steady‐state current of chronoamperometric experiments at −0.2 V (Figure S14, Supporting Information) for Au@Ag core–shell nanoparticles (blue squares) and the two nanorattle compositions, Ag_61_Au_39_ (green circles) and Ag_47_Au_53_ (red triangles), recorded at varying concentrations of HClO_4_. Notably, the nanorattles exhibit a more pronounced and systematic rise in current density with increasing electrolyte concentration than the solid Au@Ag sample. Moreover, the more highly porous Ag_47_Au_53_ nanorattles show the greatest relative increase in current density, consistent with our hypothesis that deeper pores respond more strongly to changes in EDL thickness. We attribute this enhancement to the shrinking of the EDL at higher ionic strengths, effectively making the pores available and granting reactant species fuller access to the interior catalytic sites.^[^ ^8^, ^23^ ^]^ By contrast, the core–shell architecture, lacking a prominent hollow region, is less responsive to variations in electrolyte concentration, resulting in comparatively modest gains. These observations align well with our prediction that nanoconfinement is dynamically regulated by ion‐screening effects, and they provide strong evidence that careful tuning of electrolyte composition can selectively activate or suppress catalysis in the nanoscale voids of our hollow Ag–Au architectures.^[^ ^24^ ^]^

Although the systematic rise in current densities under higher electrolyte concentrations supports a nanoconfinement‐driven enhancement, it is important to decouple this phenomenon from other structural and compositional influences. First, while increased porosity can magnify the geometric surface area, the pronounced response to electrolyte concentration changes in the nanorattles (versus only modest gains in the core–shell Au@Ag) suggests that mere surface area differences cannot fully account for the observed behavior. Second, the possibility of altered surface composition is mitigated by the fact that Au exerts minimal electroactivity under these conditions (the nanorattles should have progressively lower Ag content, which is the electroactive metal in this transformation).^[^ ^22^, ^25^ ^]^ Hence, the stronger activation of more porous nanorattles cannot be simply ascribed to higher silver content at the surface. Instead, these data suggest an interplay wherein a reduced EDL at elevated ionic strengths reveals a larger fraction of the internal catalytic interface, driving the substantial performance gains.^[^ ^23^ ^]^ Consequently, we conclude that dynamic tuning of pore accessibility, rather than differences in nominal surface area or composition, is the dominant factor behind the enhanced nitrate reduction in the nanorattle systems.

Turning to alkaline media (0.1 mol L^−1^ NaOH), we observed a similar trend of catalytic performance under dark conditions, in which the nanorattle catalysts (Ag_61_Au_39_, Ag_47_Au_53_) again surpassed the core–shell Au@Ag nanoparticles (Figure S15A, Supporting Information). Figure S15B (Supporting Information) showed that, after 30 cycles, the core‐shell is significantly deactivated, while the rattles partially maintain their activity. However, the absolute current densities were lower overall compared with those in acidic solution, indicating that nitrate reduction in alkaline media proceeds less favorably. Moreover, in contrast to the pronounced plasmonic effects seen under acidic conditions, light irradiation at 427, 525, or 640 nm did not appreciably boost the current that is summarized in Figures S15C and S16 (Supporting Information). We attribute this to a mechanistic pathway in alkaline solution that may be less sensitive to hot‐electron or localized heating effects, thereby diminishing any potential light‐driven enhancement.^[^ ^26^, ^27^ ^]^ Nonetheless, the superior activity of the nanorattles relative to their core–shell counterpart was consistently retained, suggesting that the porous, hollow design remains advantageous even when the nitrate reduction reaction proceeds via a less LSPR‐responsive route.

To gain deeper insights into the origin of the superior catalytic activity observed in the nanorattle catalysts, Density Functional Theory (DFT) calculations were performed. We constructed theoretical models representing the Au@Ag core–shell nanoparticles, as well as the Ag_61_Au_39_ and Ag_47_Au_53_ nanorattles, and fully relaxed their atomic geometries to accurately capture their structural and electronic characteristics (Figure S17, Supporting Information). We note that the slab models employed in our DFT calculations are a simplification of the 3D nanorattle morphology. Rather than reproducing the full particle geometry, which is not computationally feasible, the models were designed to capture the local electronic environment of the catalytically active shell. Galvanic replacement between Au and Ag is known to yield alloyed AgAu shell compositions, as confirmed in our catalysts by STEM–EDS mapping. Accordingly, the slab models were constructed to represent AgAu alloy surfaces with varying Ag content, approximating the experimentally observed shell compositions of Au@Ag, Ag_61_Au_39_, and Ag_47_Au_53_. This approach allows us to probe the fundamental Au–Ag synergy at the shell interface, which governs adsorption energetics, d‐band shifts, and reaction barriers, and thereby rationalizes the experimentally observed catalytic trends.

**Figure 5:**
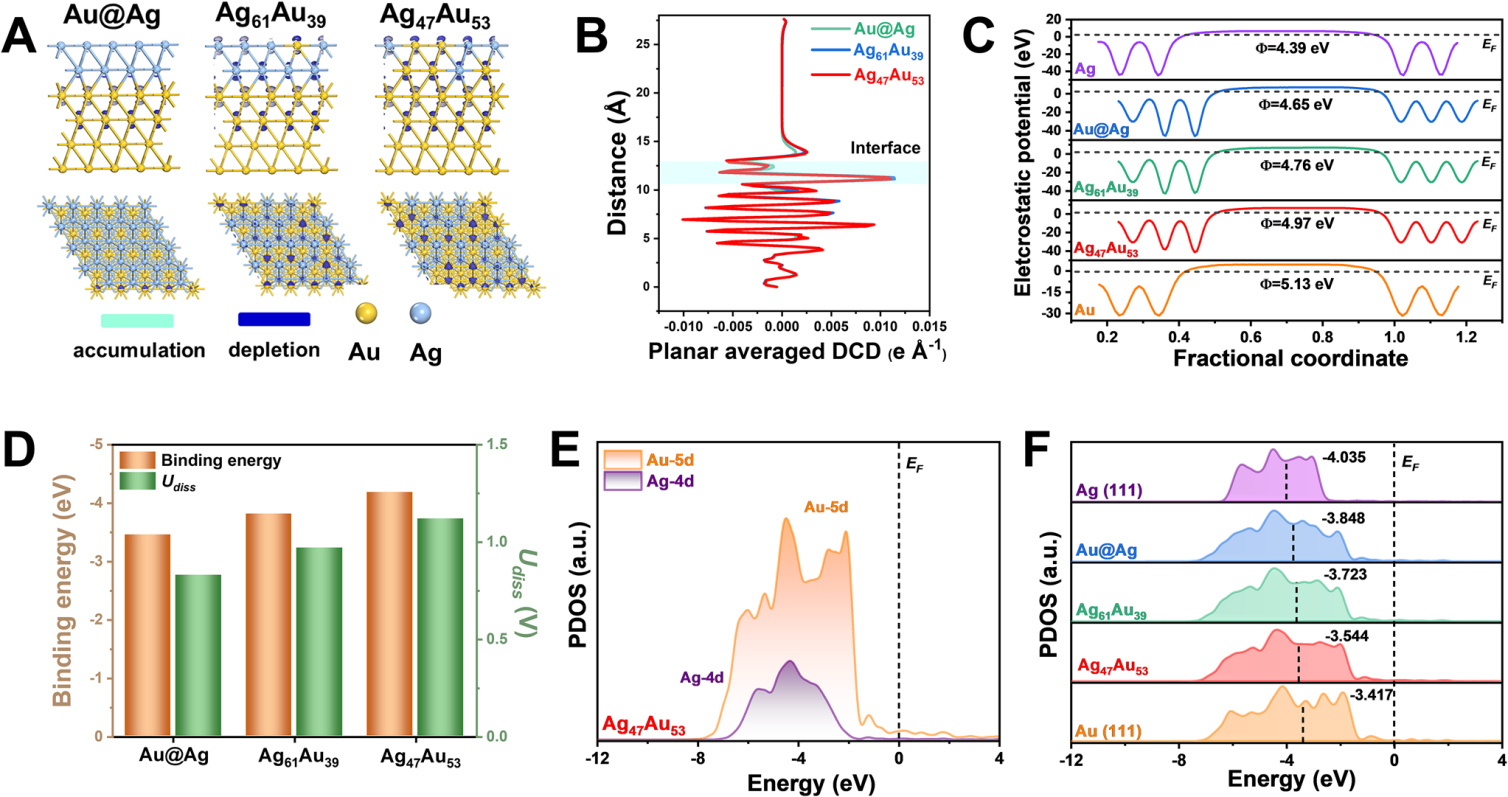
DFT analysis of electronic structure and surface energetics in core–shell and nanorattle models. A) Charge density difference plots for Au@Ag, Ag_61_Au_39_, and Ag_47_Au_53_ nanoparticle models (side and top views). Cyan and blue contours represent regions of electron accumulation and depletion, respectively. B) Plane‐averaged differential charge density (DCD) profiles across the interfaces of the models shown in (A), indicating variations in interfacial electronic structure. C) Electrostatic potential maps for Ag(111), Au@Ag, Ag_61_Au_39_, Ag_47_Au_53_, and Au(111). D) Calculated binding energy (BE) and dissolution potential (U_diss_) of surface Ag atoms across different structures. E) Projected density of states (PDOS) curves for the Ag_47_Au_53_ model, and F) comparison of Ag d‐orbital PDOS across the various models.

Charge density difference (CDD) analyses for these models (**Figure** **5**A) reveal pronounced electron redistribution at the interface in the AgAu alloy shells. Electron accumulation (cyan) and depletion (blue) regions indicate that charge rearrangement is substantially stronger in the Ag_61_Au_39_ and Ag_47_Au_53_ nanorattles compared to the solid Au@Ag nanoparticles,^[^ ^28^ ^]^ a result further supported by planar‐averaged differential charge density (DCD) profiles (Figure 5B). This significant interfacial electron redistribution is beneficial, as it facilitates efficient charge transfer processes, particularly under plasmon excitation conditions. Consistently, the electron localization function (ELF; Figure S18, Supporting Information) suggests greater electron delocalization within Ag_61_Au_39_ and Ag_47_Au_53_.^[^ ^29^ ^]^ To further elucidate electron‐transfer behavior, we examined the electrostatic potential and work function (W_F_) across different model surfaces (Figure 5C). The Au(111) surface exhibited the highest W_F_, whereas Ag(111) showed the lowest. Intermediate W_F_ values were found for all core–shell and nanorattle models, indicating spontaneous electron transfer from the Au cores toward the AgAu shells driven by their W_F_ differences.^[^ ^30^ ^]^ This built‐in charge separation, amplified under LSPR excitation, can contribute to the enhanced catalytic activity observed experimentally.

Next, we assessed the thermodynamic and electrochemical stability of Ag atoms, the active catalytic species in these systems. As shown in Figure 5D, Ag atoms in both Ag_61_Au_39_ and Ag_47_Au_53_ exhibit significantly more negative binding energies (BE) compared to those in the Au@Ag structure, signifying stronger metal–metal interactions and improved structural stability. These findings are supported by integrated crystal orbital Hamilton population (ICOHP) analyses (Figure S19, Supporting Information). Moreover, dissolution potentials (U_diss_) of Ag atoms are more positive in the nanorattles, reflecting greater electrochemical robustness under catalytic conditions.^[^ ^31^ ^]^ As Au has a more positive standard reduction potential than Ag, this agrees with the fact that Ag in AgAu alloys would be prone to oxidation. Taken together, these results provide strong evidence for enhanced stability in Ag_61_Au_39_ and Ag_47_Au_53_ nanorattles during NO_3_RR, in agreement with our experimental data.

The projected density of states (PDOS) analysis provides additional insights into the electronic interactions within these catalysts (Figure 5E,F; Figure S20A–D, Supporting Information). We observed substantial orbital overlaps between Ag 4d and Au 5d bands in all structures, indicative of efficient electron transfer pathways between the metals.^[^ ^32^ ^]^ The Ag 4d orbitals are situated deeper below the Fermi level (E_F_), thus serving as effective electron reservoirs, while the Au 5d orbitals exhibit broader and elevated positions closer to E_F_, enhancing their catalytic role in regulating adsorption and surface reactivity.^[^ ^33^ ^]^ The d‐band centers (*d*_c_) of the nanorattles shift upward with increasing Au content, suggesting fine‐tuning of adsorption strength toward reaction intermediates, especially in the highly porous Ag_47_Au_53_ structure. Such modulation in electronic structure is instrumental in promoting superior nitrate reduction activity.

**Figure 6:**
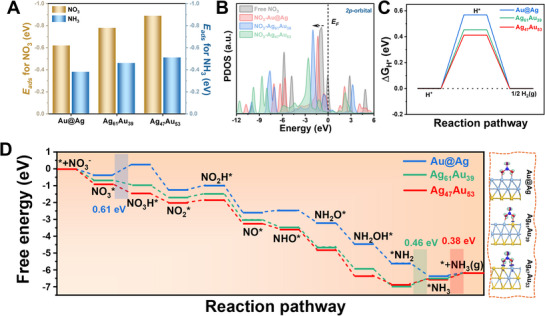
Theoretical analysis of the NO_3_RR mechanism. A) Adsorption energies of NO_3_ and NH_3_ on the surfaces of Au@Ag, Ag_61_Au_39_, and Ag_47_Au_53_ nanoparticle models. B) PDOS for the 2p orbitals of free and adsorbed NO_3_ molecules on various top sites in the three models, revealing orbital hybridization and electronic coupling with the surface. C) Calculated Gibbs free energies for hydrogen adsorption (H*) on Au@Ag, Ag_61_Au_39_, and Ag_47_Au_53_ surfaces, used to assess HER competition under NO_3_RR conditions. D) Complete Gibbs free energy diagrams for NO_3_RR over Au@Ag, Ag_61_Au_39_, and Ag_47_Au_53_ models, showing stepwise reaction energetics and identifying potential rate‐limiting steps.

To probe mechanistic details, we analyzed the adsorption energetics of NO_3_^−^ and NH_3_, as initial adsorption and final desorption steps influence the overall catalytic pathway (assuming complete conversion of NO_3_^−^ to NH_3_). Computationally optimized adsorption configurations (Figure S21, Supporting Information) reveal stronger nitrate binding (NO_3_*) on the Ag_61_Au_39_ nanorattle surfaces, as evidenced by more negative adsorption energies (**Figure** **6**A). Further, PDOS analysis of adsorbed NO_3_* species (Figure 6B) demonstrates enhanced electronic coupling with catalyst surfaces, highlighting stronger interaction and charge transfer in the Ag_47_Au_53_ model compared to Au@Ag and Ag_61_Au_39_.^[^ ^34^ ^]^

The Gibbs free energy of hydrogen adsorption (ΔG_H*_) serves as a key thermodynamic descriptor for assessing the availability of reactive H* intermediates during nitrate electroreduction. Importantly, ΔG_H*_ has been shown to correlate with the position of the *d*_c_, which influences the binding strength of intermediates (Figure 5F). As shown in Figure 6C, the Ag_47_Au_53_ nanorattles exhibit the most favorable hydrogen adsorption thermodynamics, with a ΔG_H*_ of 0.41 eV, lower than both Ag_61_Au_39_ (0.45 eV) and Au@Ag (0.57 eV). This suggests that Ag_47_Au_53_ more readily facilitates the formation of surface‐bound hydrogen species, thereby promoting key hydrogenation steps in the NO_3_RR pathway without acting as a poisoning species.^[^ ^28^ ^]^

Notably, the calculated adsorption free energies of NO_3_* are significantly more exergonic than those of H*, particularly for the Ag_61_Au_39_ (−0.67 eV) and Ag_47_Au_53_ (−0.89 eV) models (Figure 6D). This thermodynamic preference for NO_3_^−^ over hydrogen indicates strong nitrate affinity, further supported by charge density difference (CDD) maps (Figure 6D, right panels), which reveal substantial electron transfer from the surface to the adsorbed NO_3_*, highlighting the activation of the molecule. Figure 6D also presents the full Gibbs free energy diagrams for NO_3_RR on the three catalyst models. For Ag_47_Au_53_, the desorption of NH_3_ is identified as the rate‐determining step (RDS), with a relatively low energy barrier of 0.38 eV. This is significantly lower than the RDS in Au@Ag (0.61 eV, corresponding to the hydrogenation of NO_3_^−^ to NO_3_H*) and in Ag_61_Au_39_ (0.46 eV, associated with the NH_2_* to NH_3_* conversion). These findings suggest that the Ag_47_Au_53_ nanorattles strike an optimal balance between adsorption strength and product desorption, enabling efficient NO_3_RR conversion.^[^ ^35^ ^]^ Taken together, these results demonstrate that the superior catalytic activity of Ag_47_Au_53_ arises from a combination of optimized geometric configuration, modulated electronic structure, and enhanced charge dynamics under LSPR excitation. This synergy positions nanorattles with tailored porosity and composition as a promising platform for selective and efficient electrochemical nitrate reduction.

## 3 Conclusion

In this study, we demonstrated that hollow, bimetallic Au@AgAu nanorattles serve as highly effective electrocatalysts for the electrochemical NO_3_RR, outperforming their solid Au@Ag counterparts in both acidic and alkaline environments. By strategically tuning the level of porosity through partial galvanic replacement, we established that accessible pores strongly promote nanoconfinement effects, thereby enhancing catalytic activity under electrolyte conditions that modulate the electric double layer (EDL). The systematic increase in current density with rising ionic strength supports our hypothesis that thinning of the EDL effectively “opens” the nanorattles’ interior surfaces, revealing a greater fraction of the active interface confined within the hollow shell. In addition to these morphological advantages, plasmonic excitation, particularly under acidic conditions, was found to synergistically enhance activity, with light‐driven hot carriers and local heating accelerating nitrate and nitrite reduction. While alkaline media showed reduced overall activity and minimal plasmonic enhancement, the nanorattles presented higher stability when compared to the solid‐core analogues, highlighting the robustness and versatility of the nanoconfined architecture. Complementing our experimental findings, DFT calculations revealed that the electronic structure of the AgAu shell can be finely tuned by composition, leading to more favorable d‐band alignment, enhanced electron delocalization, and charge transfer between core and shell. The Ag_47_Au_53_ nanorattles exhibited optimal hydrogen and nitrate adsorption energetics, reduced reaction barriers, and improved thermodynamic selectivity toward nitrate over competing hydrogen evolution. These computational insights reinforce the conclusion that geometric confinement, interfacial electronic modulation, and plasmonic enhancement act in concert to drive efficient multi‐step catalysis. Altogether, our results crystallize the importance of a holistic design strategy that integrates nanoscale confinement, bimetallic interfaces, and electrolyte‐responsive porosity to optimize multi‐electron electrocatalytic pathways. We anticipate that similar core–shell or nanorattle frameworks could be extended to other critical transformations, such as CO_2_ reduction, oxygen evolution, or hydrogen peroxide synthesis, where dynamic tuning of the local reaction environment may unlock new levels of activity, selectivity, and long‐term catalyst durability.

## 4 Experimental Section

### Materials and Instrumentation

All reagents were used as received, without further purification. Acetone (99%, Honeywell), isopropanol (HPLC grade, 99.9%, Sigma–Aldrich), sodium citrate tribasic dihydrate (99%, Sigma–Aldrich), gold(III) chloride trihydrate (HAuCl_4_·3H_2_O, 99.9%, Sigma–Aldrich), sodium citrate dihydrate (99%, Sigma–Aldrich), silver nitrate (99%, Sigma–Aldrich), polyvinylpyrrolidone (PVP, 99%, Sigma–Aldrich), sodium hydroxide (VWR, 99.2%), ascorbic acid (99%, Sigma–Aldrich), perchloric acid (ACS reagent, 70%, Sigma–Aldrich), sodium nitrate (trace metals basis, 99.995%, Sigma–Aldrich), sodium nitrite (trace metals basis, 99.999%, Sigma–Aldrich), sodium hydroxide (98.5–100%, AnalaR NORMAPUR), Nafion perfluorinated resin aqueous dispersion (10 wt.% in water, Sigma–Aldrich), and Vulcan XC–72R carbon black (Fuel Cell Store) were employed. Deionized (DI) water (18.2 MΩ cm, Milli‐Q) was used for all experiments.

UV–vis absorption spectra were collected on a Shimadzu UV‐2600 spectrophotometer. After thorough washing of each nanomaterial (three times with ethanol, three times with hot water at 100 °C), Au, Au@Ag, Ag_61_Au_39_, and Ag_47_Au_53_ nanoparticle suspensions were dispersed in water. All spectra were recorded using water as a reference. The exact Au and Ag compositions of the catalysts were determined via microwave plasma–atomic emission spectroscopy (MP–AES) on an Agilent Technologies MP–AES 4100 instrument. Standard solutions of Au and Ag (2–10 ppm) were prepared using commercially sourced gold (III) standard solution and silver nitrate.

Field emission scanning electron microscopy (SEM) was performed on a Hitachi S‐4800. Samples were prepared by drop‐casting each nanoparticle suspension onto a Si wafer, followed by drying under ambient conditions. Transmission electron microscopy (TEM) images were obtained on a JEOL JEM‐1400 microscope operating at an accelerating voltage of 120 kV. To prepare TEM samples, an aqueous suspension of the catalysts was dripped onto a carbon film‐supported copper grid (400 square mesh). Scanning transmission electron microscopy (STEM) bright field, high‐angle annular dark‐field (HAADF) images, and EDS were obtained using a Hitachi SU9000 low kV STEM with an accelerating voltage of 30 kV. High‐resolution STEM bright field and HAADF images and STEM energy‐dispersive X‐ray spectroscopy (STEM−EDS) elemental maps were obtained with a Talos F200X transmission electron microscope. Samples for STEM/HAADF were prepared using an analogous procedure as described for TEM.

### Synthesis of Au Nanoparticles and Au@Ag Core–Shell NPs

Gold nanoparticles (Au NPs) were synthesized using the classical Turkevich method.^[^ ^36^, ^37^ ^]^ In a 250 mL round‐bottom flask, 100 mL of 0.25 mM AuCl_4_^−^ (aq) was heated to 115 °C (oil bath) with stirring for 15 min. Then, 3 mL of 1 wt.% aqueous sodium citrate dihydrate was added, and the mixture was stirred for an additional 15 min, producing a red suspension of monodisperse spherical Au NPs. Core–shell Au@Ag nanoparticles were formed via stepwise addition of Ag onto Au NPs.^[^ ^38^ ^]^ In a 25 mL round‐bottom flask, 10 mL of Au NPs was combined with 150 µL of 100 mm NaOH and 120 µL of 100 mm ascorbic acid. After 5 min stirring, 30 µL of 100 mm AgNO_3_ was added, and the mixture was allowed to react for 30 min. This step was repeated three more times until a dark orange suspension formed. All steps were carried out at room temperature.

### Synthesis of Au@AgAu Nanorattles (Ag_61_Au_39_, and Ag_47_Au_53_)

Nanorattles were prepared by galvanic replacement.^[^ ^18^ ^]^ In a 25 mL round‐bottom flask, 10 mL of 0.1 wt.% PVP (aq) and 5 mL of Au@Ag suspension were stirred at 100 °C for 10 min. Subsequently, 1 mL of 0.2 mM (Ag_61_Au_39_) or 0.4 mM (Ag_47_Au_53_) of HAuCl_4_ (aq) was added dropwise, and the mixture was stirred for an additional 10 min. The reaction was allowed to cool to room temperature, and the resulting product was washed twice with ethanol and twice with hot water (by centrifugation at 14500 rpm and removal of the supernatant). The final nanoparticle pellets were resuspended in water and briefly sonicated before characterization and use.

### Electrochemical Measurements

All electrochemical studies were performed in a three‐electrode single‐compartment glass cell. A glassy carbon rod (6 mm diameter, geometric area 0.2827 cm^2^) served as the working electrode (GCE), while a high‐area graphite rod was used as the counter electrode. Potentials are reported versus the reversible hydrogen electrode (RHE) prepared in the same supporting electrolyte (0.1 m HClO_4_ or 0.1 m NaOH). Electrolytes were made by diluting ultrapure HClO_4_ or dissolving NaOH pellets in 18.2 MΩ cm water. The GCE was polished with alumina slurry, followed by sonication (5 min each) in DI water and acetone. After cleaning, 1 mL of each nanoparticle suspension (Au@Ag or Au@AgAu) was washed three times with ethanol and three times with hot water (100 °C). A catalyst ink was prepared by dispersing the nanoparticles in a water:isopropanol mixture with Nafion (10 wt.%), plus Vulcan XC–72R carbon black. Then, 30 µL of this ink was drop‐cast onto the GCE and dried in air at 60 °C for 1 h. The Ag loading was 97.5, 42.7, and 32.8 µg cm^−^^2^ for Au@Ag, Ag_61_Au_39_, and Ag_47_Au_53_, respectively, and the Vulcan carbon loading was 100 µg cm^−^^2^. All electrochemical measurements were performed at 25 °C using an Autolab PGSTAT 128 N potentiostat with Scan 250 module. Before each measurement, the electrolyte was purged with Ar gas (2.2 grade), and Ar flow was maintained over the solution during data collection. To compare catalysts fairly, cyclic voltammograms (CVs) were normalized by the Ag mass in each sample. Corresponding CVs normalized by geometric area are presented in the Supplementary Information (Figure S6, Supporting Information).

For NO_3_RR studies, NaNO_3_ or NaNO_2_ was added to the electrolyte from a 1 M stock solution to achieve the desired concentration. CV and linear scan voltammetry (LSV) measurements were conducted at 10 mV s^−1^. Chronoamperometric (CA) tests were performed at −0.2 V (vs RHE), varying the perchloric acid concentration to investigate the role of pore structure and catalyst stability. The cell temperature was controlled at 25 °C with a Julabo F12–MA refrigerated circulator. Plasmonic excitation was provided by a Kessil PR160L LED lamp at 427, 525, or 640 nm (≈60 mW cm^−^^2^). For NO–saturated adlayers, the GCE was immersed in a 0.1 m HClO_4_ solution containing 0.01 m NaNO_2_ for 3 min, then rinsed with DI water. NO stripping was performed at 2 mV s^−1^ from 0.4 V in 0.1 m HClO_4_. Photoexcitation of these adlayers probed NO removal and synergy with LSPR at varying potentials.

### Theoretical Simulations

DFT calculations were performed with the first‐principles simulation Cambridge Sequential Total Energy Package (CASTEP) module in Materials Studio software.^[^ ^39^ ^]^ The exchange‐correlation potential was described by the generalized gradient approximation (GGA) with the Perdew‐Burke‐Ernzerhof (PBE) functional.^[^ ^40^ ^]^ The interactions between valence electrons and ionic cores were described by the OTFG ultrasoft pseudo‐potential method. A plane‐wave basis set with a cutoff energy of 400 eV was assigned to the potential method. The empirical dispersion correction in Grimme's scheme was employed to consider the van der Waals (vdW) interaction. The Broyden‐Fletcher‐Goldfarb‐Shannon (BFGS) algorithm with a medium quality setting of k‐points was used for all the energy minimizations in this work. The geometry optimization convergence tolerances for the energy change, maximum force, and maximum displacement were 5 × 10^−5^ eV/atom, 0.001 eV/Å, and 0.005 Å, respectively. For all the models, a 20 Å vacuum space was set in the z‐axis to guarantee full relaxation. The free energy (ΔG) calculations of each reaction step are based on the standard hydrogen electrode model. The reaction free energy change is calculated by the following equation:^[^ ^41^ ^]^

(1) $$\Delta G=\Delta E+\Delta E_{\mathrm{ZPE}}-T\Delta S$$

where ΔE is the total energy difference before and after intermediate adsorption, ΔE_ZPE_ and ∆S are the differences of zero‐point energy and entropy, respectively.

## Conflict of Interest

The authors declare no conflict of interest.

## Supporting information



Supporting Information

## Data Availability

The data that support the findings of this study are available in the supplementary material of this article.
