# Supplementary material for: Ion‐Gated Nanoconfinement in Bimetallic Nanorattles Unlocks Enhanced Plasmonic Nitrate Reduction Electrocatalysis
Source: Small. 2025 Sep 12;21(43):e07525. doi: 10.1002/smll.202507525 (PMC12571211; doi:10.1002/smll.202507525)
Supplement: Supplementary file 1 — Supporting Information [file SMLL-21-e07525-s001.pdf]

NANO · MICRO
small

Supporting Information

for *Small*, DOI 10.1002/smll.202507525

Ion-Gated Nanoconfinement in Bimetallic Nanorattles Unlocks Enhanced Plasmonic Nitrate Reduction Electrocatalysis

*Flavia G. da Silva, Kaline N. da Silva, Shiqi Wang, Hugo L. S. Santos, Eric V. Formo and Pedro H. C. Camargo**

Supporting Information for

Ion-Gated Nanoconfinement in Bimetallic Nanorattles Unlocks Enhanced Plasmonic Nitrate Reduction Electrocatalysis

Flavia G. da Silva,[a,†] Kaline N. da Silva,[a,†] Shiqi Wang,[a] Hugo L. S. Santos,[a] Eric V. Formo,[b] and Pedro H. C. Camargo[a,*]

[a]*University of Helsinki, Department of Chemistry, A.I. Virtasen aukio 1, Helsinki, Finland*

[b]*University of Georgia, Georgia Electron Microscopy, Athens, Georgia 30602, USA*

**Corresponding author. Email: pedro.camargo@helsinki.fi*

[†]*These two authors contributed equally to this work.*

Table S1. Catalysts' elemental composition from ICP–AES analysis. Elemental contents of Au and Ag were measured by inductively coupled plasma–atomic emission spectroscopy (MP–AES) to determine the bulk ratios in each sample. These data confirm the intended compositional variations arising from the galvanic replacement protocol, thereby linking the extent of Au incorporation to the resultant porosity and nanorattle formation.

	**Au at. %**	**Ag at. %**	**Ag/Au ratio**
**Au@Ag**	15	85	5.7
**$Ag_{61}Au_{39}$**	39	61	1.6
**$Ag_{47}Au_{53}$**	53	47	0.9

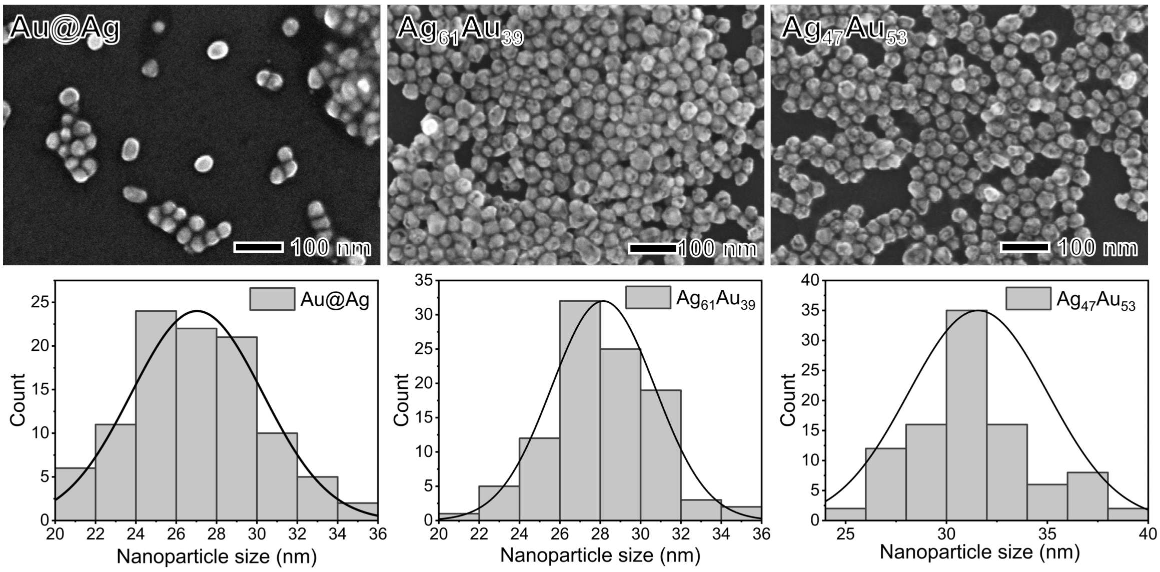

Figure S1. SEM images and size distributions of Au@Ag core–shell nanoparticles and nanorattles. (A) SEM images of Au@Ag core–shell nanoparticles, (B) $Ag_{61}Au_{39}$, and (C) $Ag_{47}Au_{53}$ nanorattles. Insets show the corresponding size distribution histograms, confirming well-defined morphology and size control.

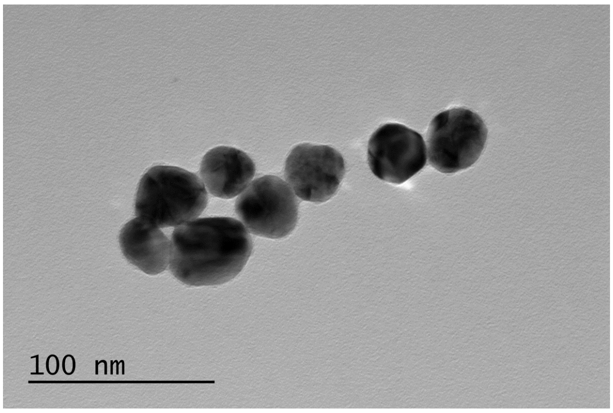

Figure S2. TEM images of Au@Ag core–shell nanoparticles. TEM images of Au@Ag core–shell nanoparticles prior to galvanic replacement, showing smooth and dense shells, in contrast to the porous structures observed in nanorattles.

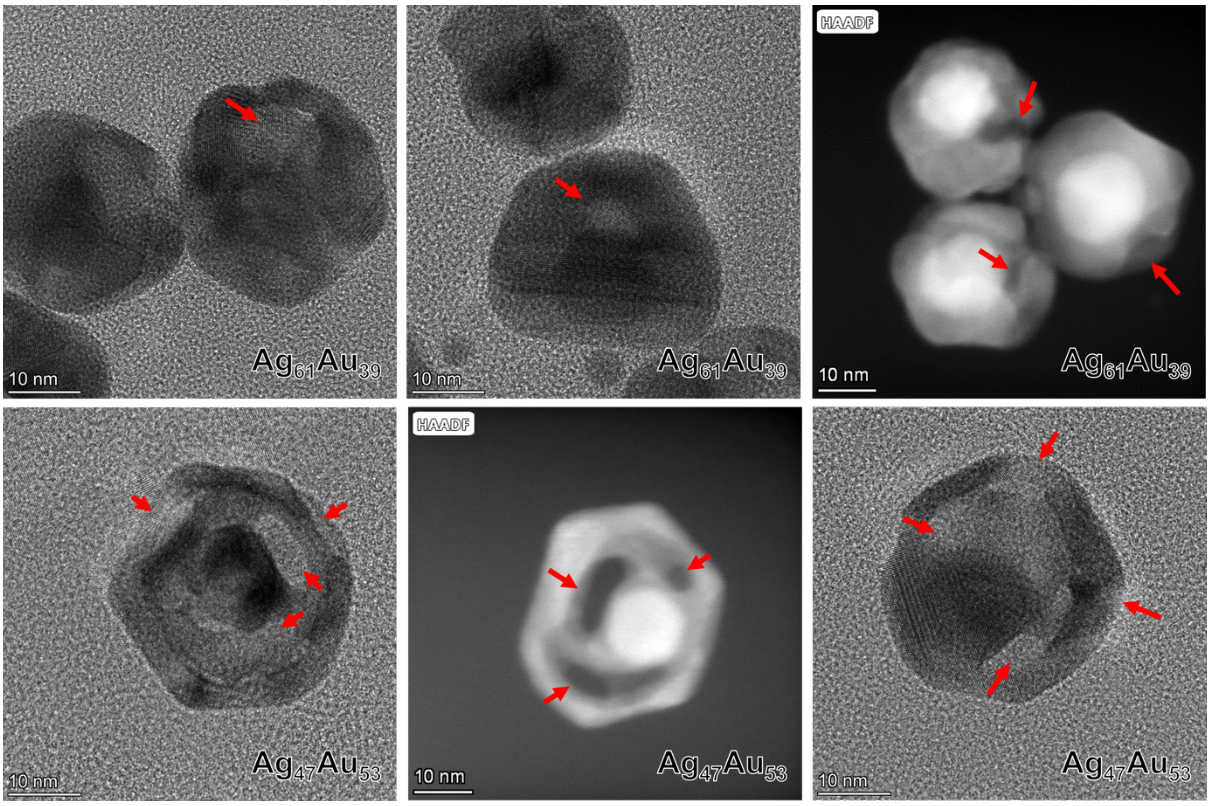

Figure S3. HRTEM images of nanorattles highlighting shell porosity. High-resolution transmission electron microscopy (HRTEM) images of $Ag_{61}Au_{39}$ and $Ag_{47}Au_{53}$ nanorattles, demonstrating the presence of nanoscale pores in the shell structure, which contribute to enhanced nanoconfinement effects

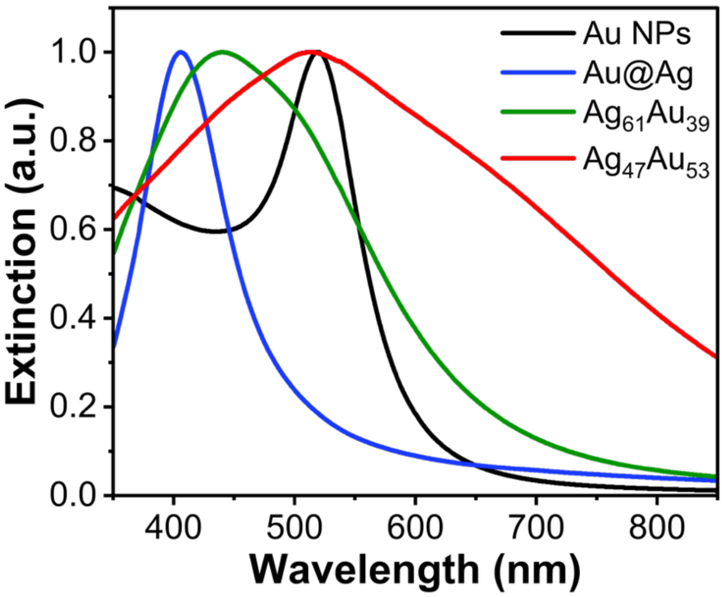

Figure S4. Optical absorption properties of Au@Ag and nanorattles. Normalized UV–Vis extinction spectra recorded in aqueous suspension for Au nanoparticles (black trace), Au@Ag core–shell nanoparticles (blue trace), $Ag_{61}Au_{39}$ nanorattles (green trace), and $Ag_{47}Au_{53}$ nanorattles (red trace). The spectral shifts reflect compositional changes and hollowing effects due to galvanic replacement.

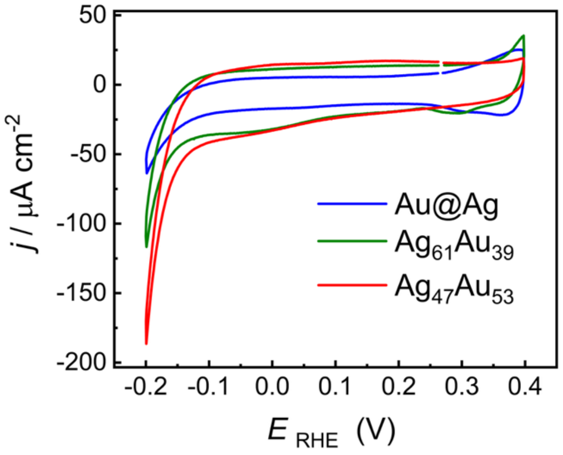

Figure S5. Voltammograms in acidic media. Cyclic voltammograms recorded in 0.1 M $HClO_4$ on Au@Ag (blue), $Ag_{61}Au_{39}$ (green), and $Ag_{47}Au_{53}$ (red) at 10 mV s^{-1} and 25°C, showing distinct electrocatalytic behavior across the different structures.

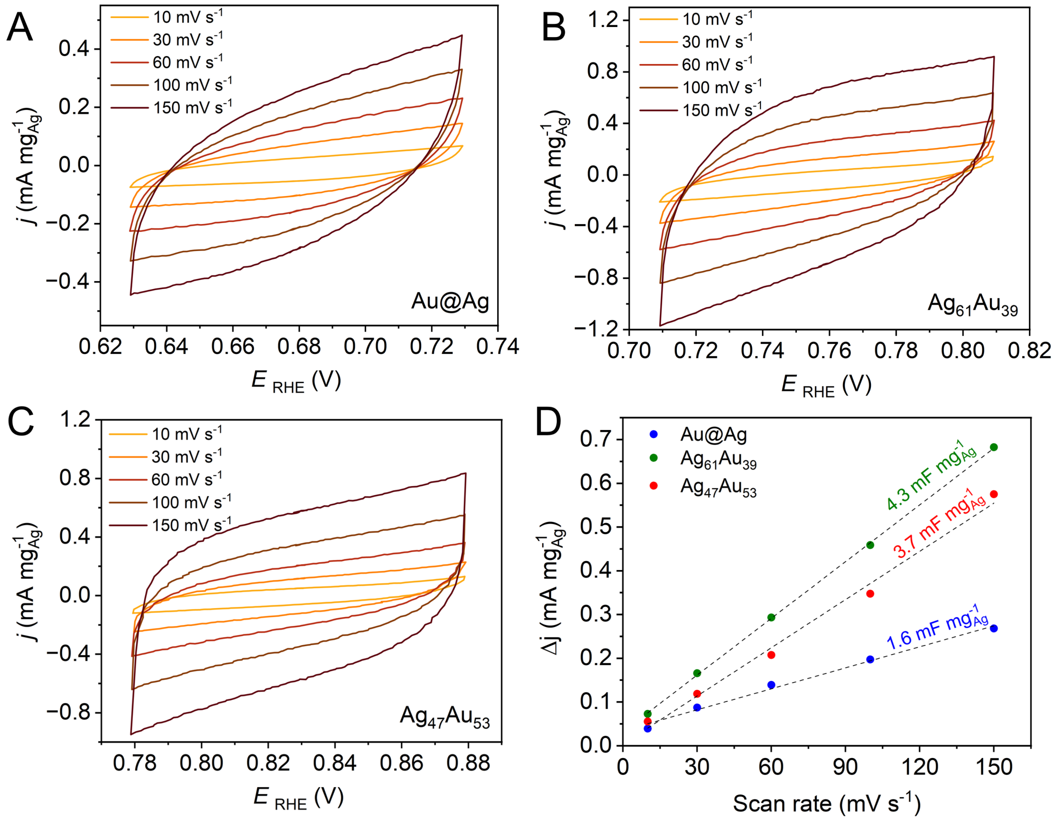

Figure S6. Double-layer capacitance measurements for Au@Ag and nanorattle catalysts. Mass-normalized cyclic voltammograms recorded in the non-faradaic region at various scan rates (10, 30, 60, 100, and 150 mV s^{-1}) for (A) Au@Ag, (B) $Ag_{61}Au_{39}$, and (C) $Ag_{47}Au_{53}$ catalysts in Ar-saturated 0.1 mol L^{-1} $HClO_4$. (D) Corresponding plots of $\Delta j = (j_{anodic} - j_{cathodic})/2$ versus scan rate, used to extract the double-layer capacitance (C_{dl}) for each catalyst. The nanorattles $Ag_{61}Au_{39}$ and $Ag_{47}Au_{53}$ exhibit significantly higher C_{dl} values (4.3 and 3.7 mF mg^{-1}, respectively) compared to Ag@Au (1.6 mF mg^{-1}), indicating a substantially larger electrochemically active surface area due to their hollow and porous architecture.

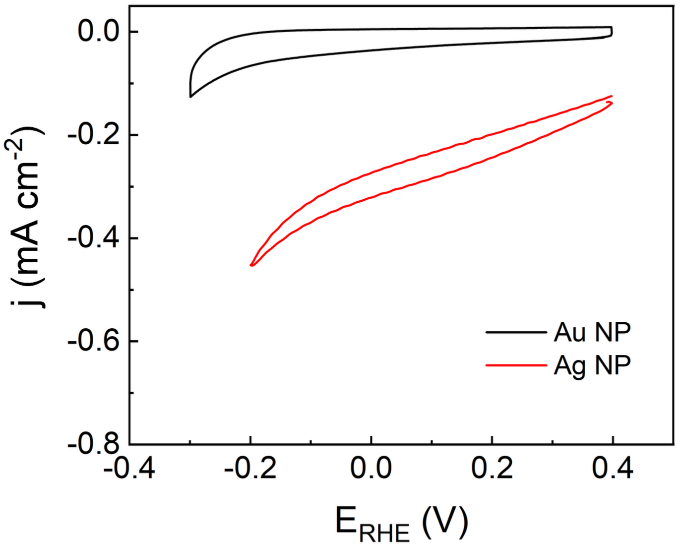

Figure S7. Control experiments with pure Au and Ag nanoparticles for NO_3RR. Cyclic voltammograms of Au nanoparticles (black) and Ag nanoparticles (red) recorded in 0.1 M $HClO_4$ containing 10 mM $NaNO_3$ at a scan rate of 10 mV s^{-1} and 25 °C. The comparison highlights the negligible NO_3RR activity of Au nanoparticles under these conditions, in contrast to the measurable activity of Ag nanoparticles, which is still lower than that of the bimetallic Au@Ag and nanorattle catalysts.

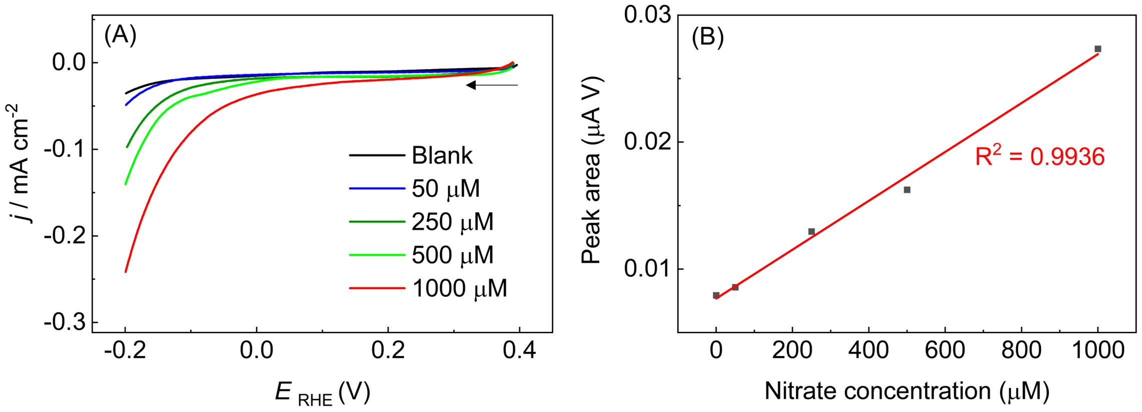

Figure S8. Nitrate concentration dependence in acidic media. (A) Reverse-scan voltammograms in 0.1 M $HClO_4$ with incremental additions of $NaNO_3$ on $Ag_{47}Au_{53}$ nanorattles at 10 mV s^{-1} and 25°C. (B) Linear dependence of reduction peak area on nitrate concentration, demonstrating the high sensitivity of the nanorattles to nitrate concentration changes.

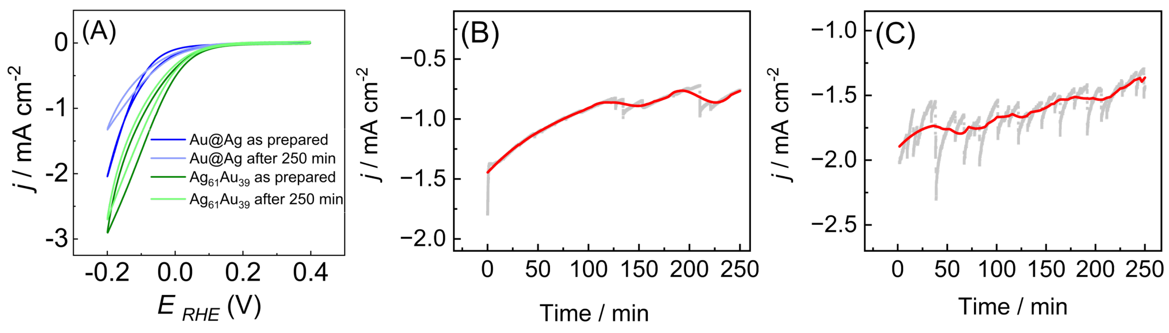

Figure S9. Comparative electrocatalytic performance of nanorattles and core–shell nanoparticles. (A) Cyclic voltammograms in 0.1 M $HClO_4$ + 10 mM $NaNO_3$ on Au@Ag (blue) and $Ag_{47}Au_{53}$ (green) at 10 mV s^{-1}. (B)Au@Ag and (C) $Ag_{47}Au_{53}$ chronoamperometric experiments at −0.2 V (vs. RHE) in the same electrolyte conditions. All measurements were conducted at 25°C.

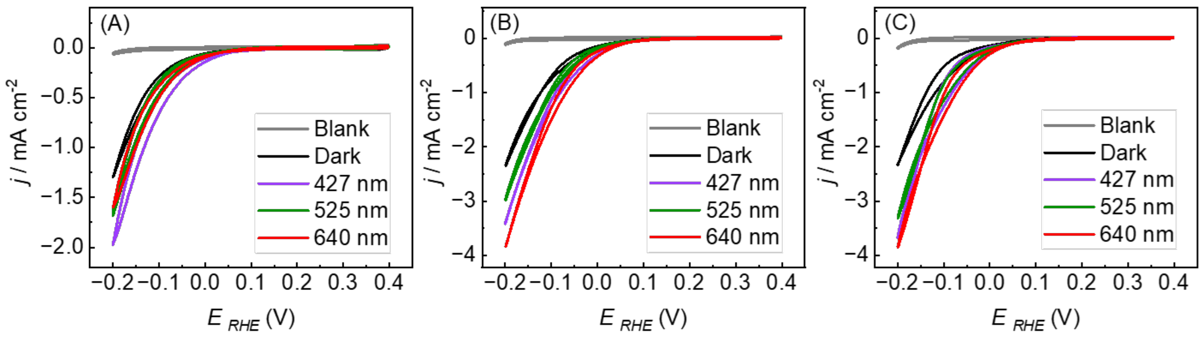

Figure S10. Light-enhanced NO_3RR in acidic media. Cyclic voltammograms in 0.1 M $HClO_4$ (dotted black line) with 10 mM $NaNO_3$ on (A) Au@Ag, (B) $Ag_{61}Au_{39}$, and (C) $Ag_{47}Au_{53}$ in dark conditions (black) and under irradiation at 427 nm (purple), 525 nm (green), and 640 nm (red). All experiments were performed at 0.01 V s^{-1} and 25°C.

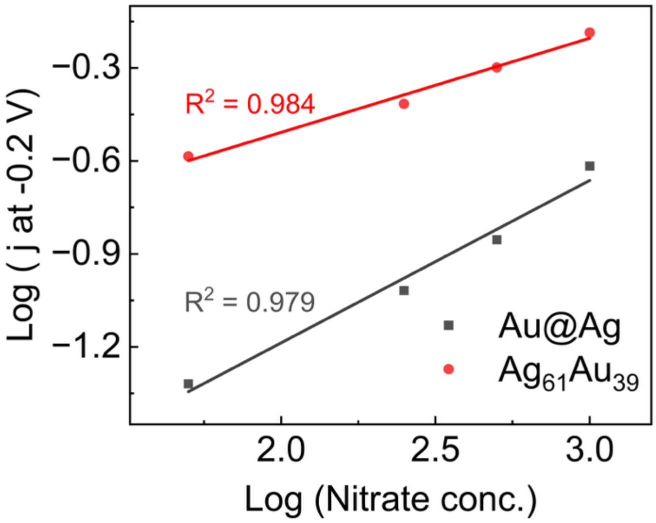

Figure S11. Nitrate concentration dependence from reverse-scan voltammetry in acidic media. Reverse-scan voltammograms in 0.1 M $HClO_4$ with stepwise $NaNO_3$ additions at 10 mV s^{-1} and 25°C, performed on Au@Ag (gray) and $Ag_{47}Au_{53}$ (red).

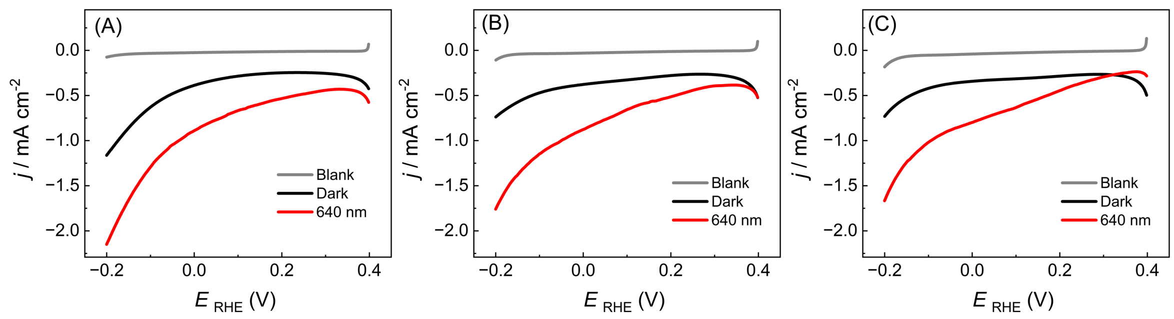

Figure S12. Nitrite reduction on Au@Ag and nanorattles in acidic media. Linear scan voltammograms in 0.1 M $HClO_4$ with 10 mM $NaNO_2$ at 10 mV s^{-1} and 25°C for (A) Au@Ag, (B) $Ag_{61}Au_{39}$, and (C) $Ag_{47}Au_{53}$, revealing distinct electrocatalytic behavior toward nitrite reduction.

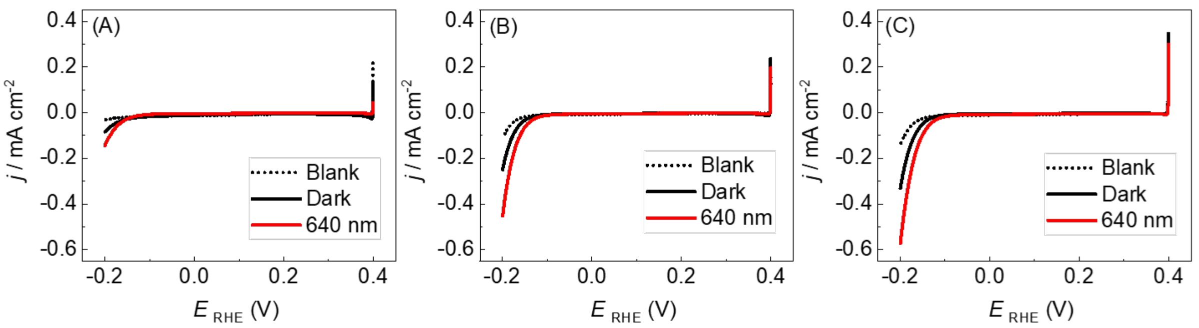

Figure S13. NO-stripping voltammograms for Au@Ag and nanorattles. NO adlayers were prepared ex-situ in 0.1 M $HClO_4$ solution containing 0.01 M $NaNO_2$ for 3 min and subsequently stripped at 2 mV s^{-1} and 25°C. (A) Au@Ag, (B) $Ag_{61}Au_{39}$, and (C) $Ag_{47}Au_{53}$. These data confirm that NO reduction occurs at more negative potentials and is unlikely to contribute significantly to observed catalytic currents.

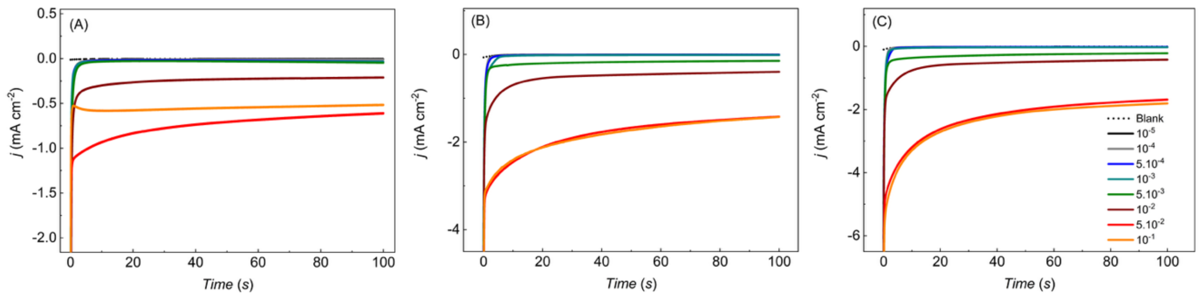

Figure S14. Electrolyte-gated nanoconfinement from chronoamperometry experiments. Chronoamperometric profiles at −0.2 V vs. RHE on (A) Au@Ag, (B) $Ag_{61}Au_{39}$, and (C) $Ag_{47}Au_{53}$, as the $HClO_4$ concentration is increased stepwise from 10^{-5} to 10^{-1} M with 10 mM $NaNO_3$, at 25°C. The systematic increase in current density with higher electrolyte concentration highlights the tunability of nanoconfinement effects.

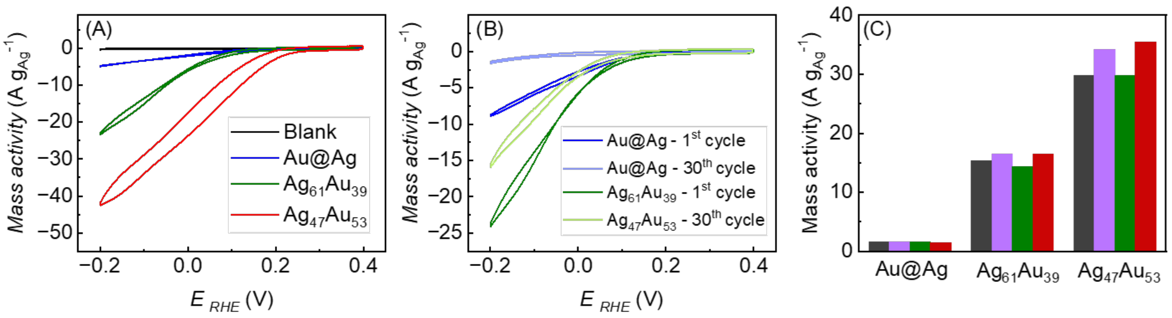

Figure S15. Nitrate reduction under alkaline conditions. (A) Cyclic voltammograms in 0.1 M NaOH (black line) with 10 mM $NaNO_3$ on Au@Ag (blue), $Ag_{61}Au_{39}$ (green), and $Ag_{47}Au_{53}$ (red) at 0.01 V s^{-1} under dark conditions at 25°C. (B) Overlaid 1st (solid) and 30th (faded) cycles for the catalysts, illustrating stability and potential changes over prolonged operation. (C) Mass activities at −0.2 V, comparing dark (black) and illuminated conditions (427 nm, purple; 525 nm, green; 640 nm, red). Under alkaline conditions, light-induced enhancements are negligible. Data collected after 30 potential cycles confirms the absence of significant plasmonic enhancement in alkaline media while demonstrating the retained electrocatalytic performance of the nanorattle structures.

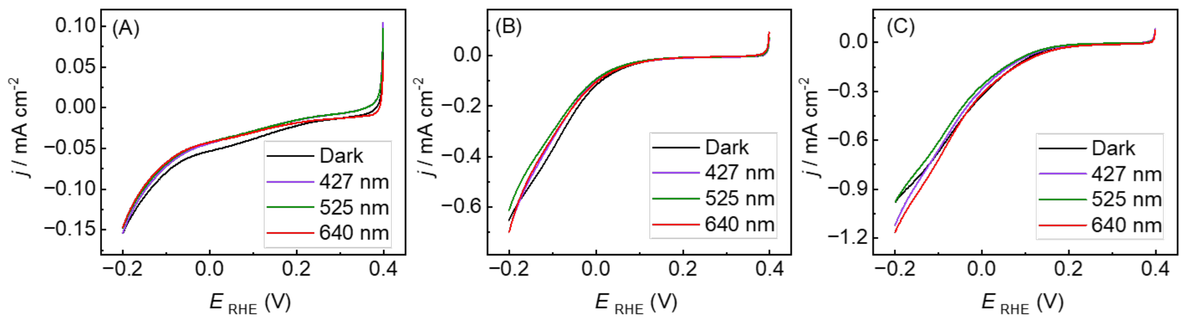

Figure S16. Plasmonic effect after 30 cycles in alkaline medium. (A–C) Voltammetric profiles recorded at 0.01 V s^{-1} and 25°C in 0.1 M NaOH containing 10 mM $NaNO_3$ for Au@Ag (A), $Ag_{61}Au_{39}$ (B), and $Ag_{47}Au_{53}$ (C), in dark conditions (black) and under LED irradiation at 427 nm (purple), 525 nm (green), and 640 nm (red).

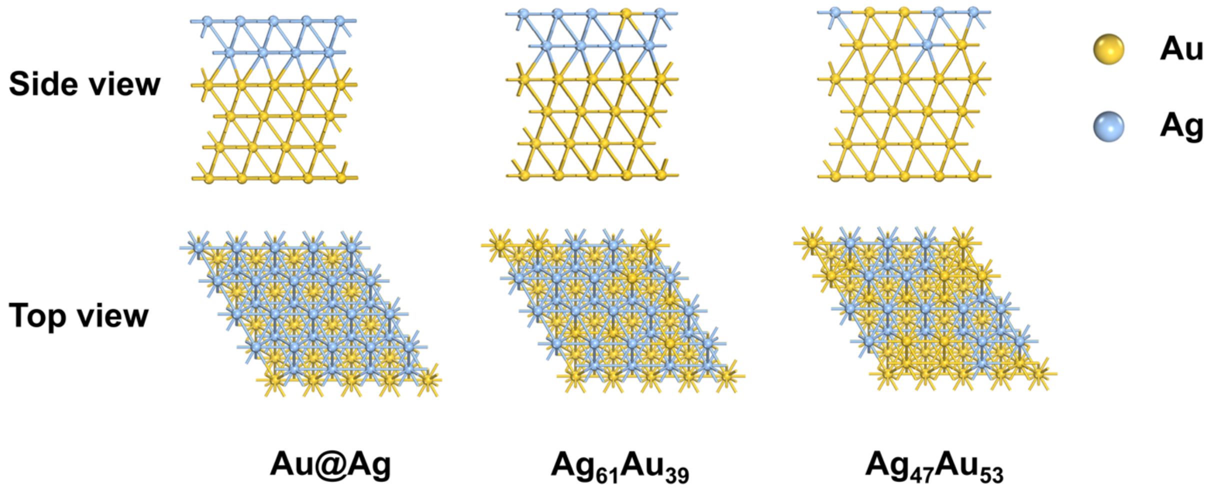

Figure S17. DFT structural models. Structural models for Au@Ag, $Ag_{61}Au_{39}$, and $Ag_{47}Au_{53}$ NPs from top and side views.

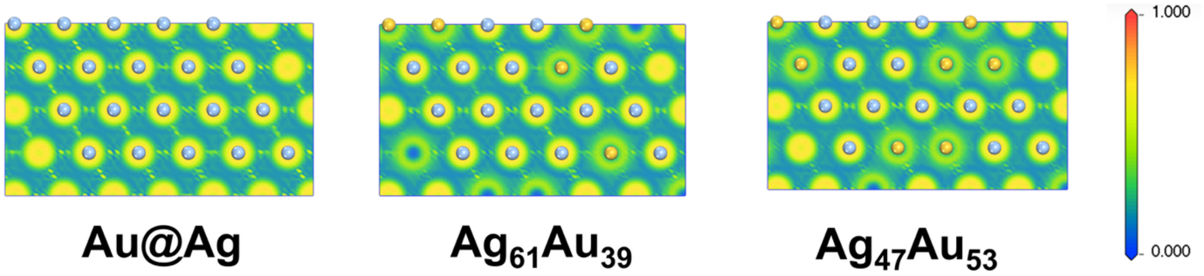

Figure S18. Electron localization function (ELF) analysis of Au@Ag and nanorattle models. Two-dimensional ELF plots for Au@Ag, $Ag_{61}Au_{39}$, and $Ag_{47}Au_{53}$ models, with ELF values ranging from 0 to 1. ELF = 0 denotes complete electron delocalization and absence of bonding, ELF = 0.5 corresponds to electron–gas-like behavior with typical Pauli repulsion, and ELF = 1 indicates strong electron localization between adjacent atoms. The progressive changes in ELF distribution across the models provide insights into bonding characteristics and electronic structure variations induced by galvanic replacement.

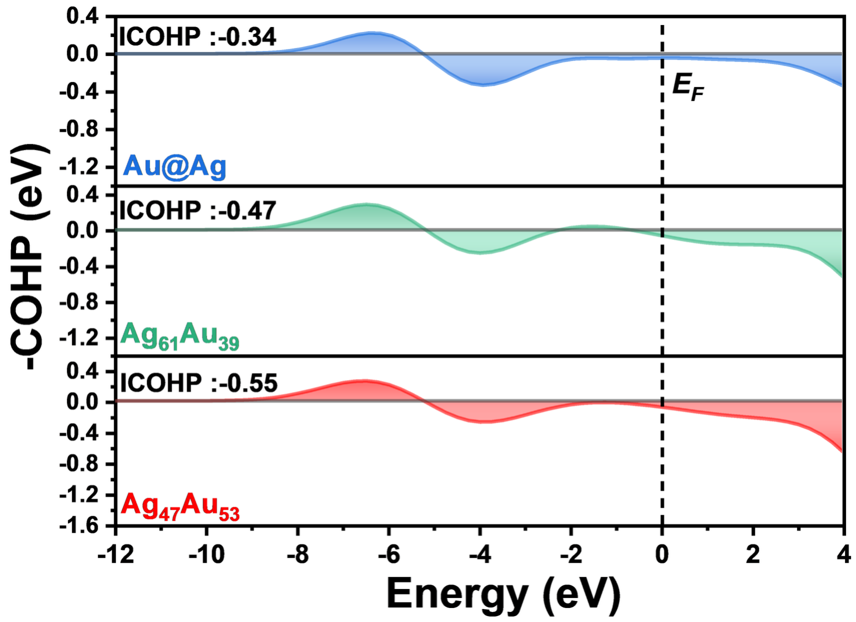

Figure S19. Integrated crystal orbital Hamilton population (ICOHP) analysis of Ag–ligand bonding interactions. ICOHP values for Ag atoms coordinated to ligand species across different surface configurations of the Au@Ag, $Ag_{61}Au_{39}$, and (C) $Ag_{47}Au_{53}$ models. The data quantify the bonding strength between Ag and adsorbed species, with more negative ICOHP values indicating stronger bonding. Differences in ICOHP trends highlight the influence of composition and nanostructure on electronic interactions at the catalytic interface.

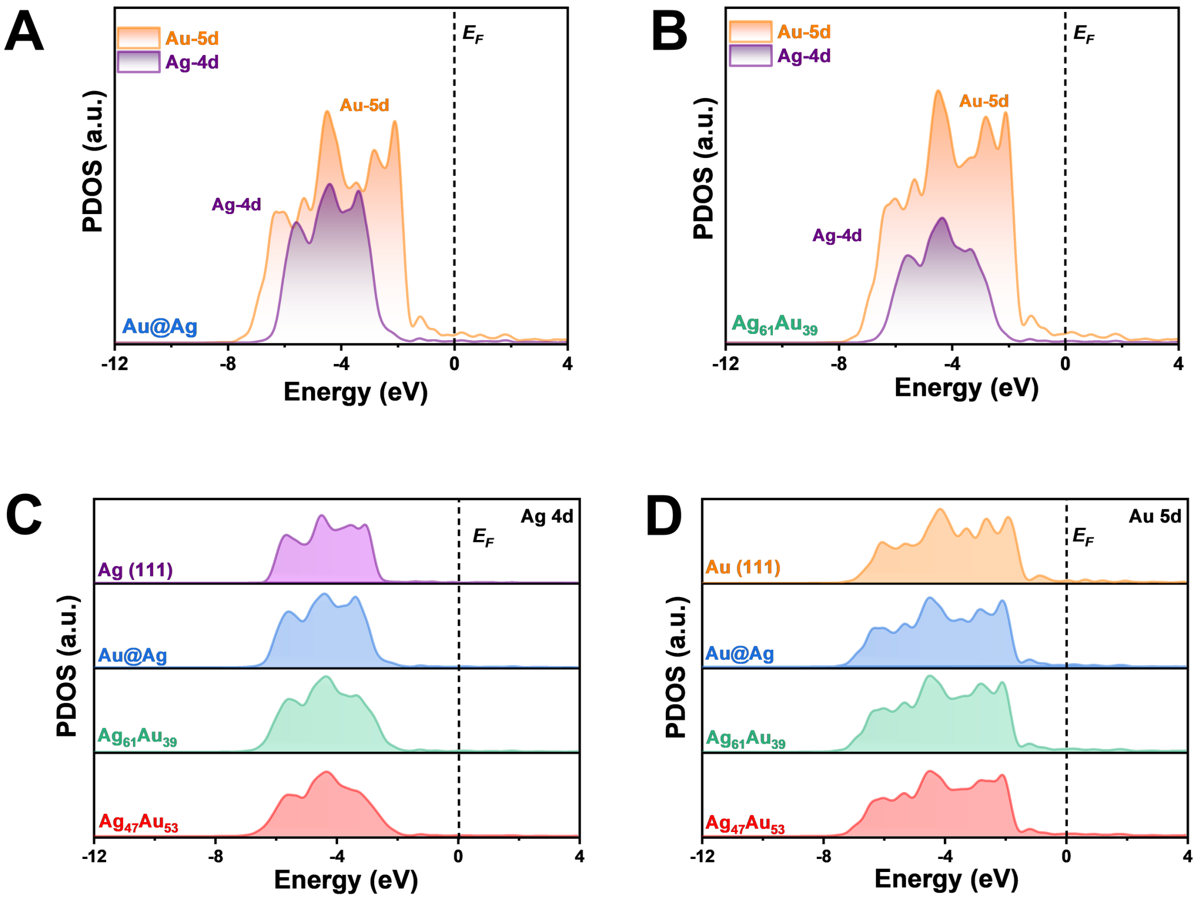

Figure S20. Projected density of states (PDOS) analysis of Au@Ag and $Ag_{47}Au_{53}$ models. (A,B) Total PDOS profiles for the Au@Ag and $Ag_{47}Au_{53}$ models. (C) Comparison of Ag 4d orbitals across the three models. (D) Comparison of Au 5d orbitals. The shifts in the d-band center and orbital density illustrate electronic modulation resulting from increased Au incorporation and structural hollowing, which impact adsorption behavior and catalytic activity. PDOS curves for (A) Au@Ag and (B) $Ag_{47}Au_{53}$ models. PDOS of (C) Ag-4d and (D) Au-5d orbitals on various models.

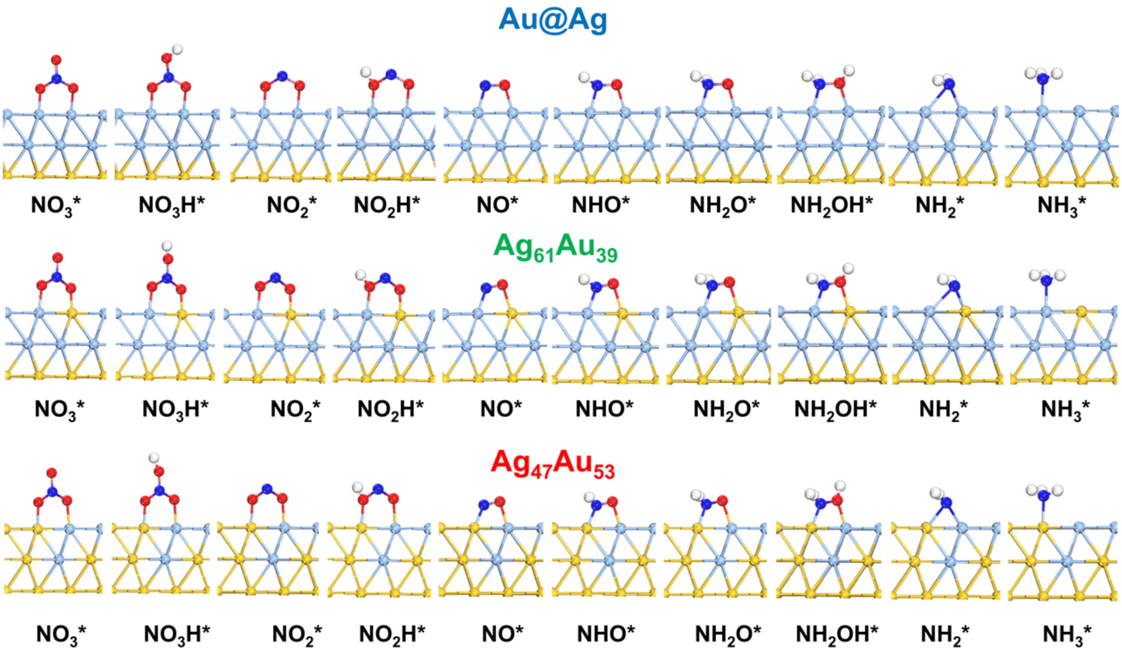

Figure S21. Optimized adsorption geometries of key NO_3RR intermediates. Adsorption configurations of nitrate reduction intermediates on Au@Ag, $Ag_{61}Au_{39}$, and $Ag_{47}Au_{53}$ surface models. Representative snapshots depict preferred binding sites and orientations of species such as NO_3^-, NO_2^-, and *NO, highlighting how structural and electronic variations influence adsorption energies and mechanistic pathways during NO_3RR.
